# Calcium and Cholecalciferol Levels in Late-Phase Laying Hens: Effects on Productive Traits, Egg Quality, Blood Biochemistry, and Immune Responses

**DOI:** 10.3389/fvets.2020.00389

**Published:** 2020-07-30

**Authors:** Youssef A. Attia, Mohammed A. Al-Harthi, Hayam M. Abo El-Maaty

**Affiliations:** ^1^Department of Agriculture, Faculty of Environmental Sciences, King Abdulaziz University, Jeddah, Saudi Arabia; ^2^Poultry Production Department, Faculty of Agriculture, Mansoura University, Mansoura, Egypt

**Keywords:** laying hens, calcium, VD_3_, egg quality, immunological responses, blood biochemistry, electron microscope

## Abstract

Productive traits and immunity in laying hens decrease sharply during the late phase of laying due to aging, which negatively affects the metabolism and hormonal status of the animals. The influence of Ca levels (3.5, 4.0, and 4.5%) and/or cholecalciferol [Vitamin D_3_ (VD_3_)] supplementation (800-, 1,000-, and 1,200-IU/kg diet or as total of 3,800, 4,000, and 4,200 IC VD_3_) on performance, egg quality, blood biochemistry, and immunity of brown egg layers was investigated. Three hundred and sixty H&N Brown egg layers (60 weeks old) were allocated at random into nine nutritional treatments of five replications (cages) of eight hens each. The control diet in this experiment contained a 3.5% Ca level with 800 IU VD_3_. The addition of VD_3_ at 1,000 and 1,200 IU to 3.5 and 4% Ca diets significantly (*P* ≤ 0.05) increased the rate of laying, egg mass, and feed conversion ratio (FCR) compared to the control diet on 3.5% and 800 U of VD_3_. Besides this, the addition of VD_3_ at 800 and 1,200 IU to 3.5% Ca level diets enhanced the Haugh unit score. Similar results were observed in eggshell quality measurements and tibia ash. Increasing the Ca concentration from 3.5 to 4 and 4.5% and increasing VD_3_ levels from 800 to 1,000 or 1,200 IU significantly and similarly increased serum total protein and globulin. In addition, VD_3_ at 1,000 IU increased serum albumin, compared to 800 IU. Increasing Ca level increased IgA, and 4 and 4.5% Ca levels similarly increased IgG and α-2 globulin compared to the 3.5% Ca diet. VD_3_ addition at 1,200 IU to the 4% Ca diet significantly increased γ-globulin compared to 1,000 IU, but decreased β-globulin. Increasing the Ca level to 4% significantly reduced serum triglycerides, and the very low-density lipoprotein and the triglyceride/high-density lipoprotein ratio were both decreased with 4 and 4.5% Ca level diets. Increasing the Ca level caused a stepwise increase in catalase, which was markedly increased with VD_3_ supplementation at 1,200 IU. Plasma estrogen was increased considerably with VD_3_ supplementation at 3.5% Ca, but parathyroid hormone levels were not affected. In conclusion, increasing Ca levels in the diet of laying hens to 4% during the late production phase could be a useful tool to improve laying performance, eggshell quality, Haugh unit score, and physiological and immunological status. Besides, VD_3_ at a 1,000 IU/kg diet to 3.5% Ca improved performance of hens fed 3.5% Ca, showing that the potential impact of VD_3_ depends on Ca concentrations.

## Introduction

Globally, both eggs and meat are acceptable and economical sources of protein for human nutrition (1). Modern hybrids of laying hens produce more than 320 eggs per year. The eggs contain 10% shell, and eggshell contains 40% Ca; thus, hens require a considerable amount of Ca for eggshell formation. Eggshell protects the eggs and is responsible for maintaining their inner quality from oviposition until use by the consumers (2). Obviosuly, egg quality and particularly shell quality decline with the age of laying hens during the late phase (60 wk or older) of production (3, 4). The decrease in eggshell quality during the late stage of production could be determined by an increase in egg size, reductions in nutrient metabolism, mainly of Ca, and in reproductive hormones, especially estrogens (5). Calcium is a critical constituent in the nutrition of laying hens, and its availability depends on dietary intestinal absorption. Still, the skeleton is an essential Ca source during the night when intestinal absorption has ceased (5, 6). Increased breakage and loss of eggs are the greatest problems in the table egg industry, causing substantial economic loss, possibly due to low egg shell quality (6, 7). Not only low eggshell quality but also loss of bone strength occurs during the late laying period (5, 8). Recent evidence has shown that proteins are integrated in the regulation of proteins driving the calcification of shell quality and function in laying hens (9, 10).

Adequate Ca consumption by laying hens is critical for ensuring reliable eggshell quality. However, the impact of dietary Ca level on egg production and quality are contradictory in the literature, and some data were reported several decades ago. Besides, there has been a significant improvement in the laying rate due to genetic progress and the use of biotechnical tools in animal breeding, together with consumers' awareness of egg quality, that needs further attention when producing functional eggs (5, 11). In the literature, Ca requirements for laying hens depend on age, production phase, environmental temperature, strain, and the concentrations of Ca, P, and vitamin D (VD) in the diet (5). For example, qualitative traits of egg and tibia weight of laying hens fed 3.5% Ca (3.77 g/d) were higher than those fed 3.0% (3.29 g/d), but increasing Ca level to 4% (4.31 g/d) did not affect eggshell quality or tibia weight (12). Besides, a daily Ca requirement of 3.75 g was recommended more than two decades ago (13, 14). On the other hand, the optimum percentage of Ca for eggshell formation was cited to be 4.73% (15). Recent guidelines of breeder companies recommend a daily Ca intake of 4.10 g during the early egg production phase (19 wk), and this gradually increases with age to reach 4.4 g% for Lohaman Brown-Classic layers in the late phase of production at 65 wk or later (16). On the other hand, the NRC (17) recommends a daily intake of 3.75 g for white and brown eggshell layers.

Cholecalciferol (VD_3_) and its two metabolites are essential for the calcification of eggshell and bone and have both hormonal and immune influences (3, 4, 18). VD_3_ can be formed in the skin of the dermis and epidermis from 7-dihydrocholesterol under ultraviolet light or can be offered as a dietary component (3, 19). Nowadays, laying hens are reared under the extensive system and housed in closed-housing in cages for different perspectives, such as the increasing intensity of production, farming profits, production of clean eggs, and improving rearing conditions. Hens housed under these conditions are not exposed to adequate natural light to transform 7-dihydrocholesterol at levels appropriate for the sufficient synthesis of VD_3_. Thus, VD_3_ is usually added to layer feeds; these are critical for the homeostasis of calcium and to maintain laying performance, bone calcification, and eggshell formation (5, 14, 20). The biosynthesis of the active form 1, 25-dihydroxycholecalciferol (1,25-(OH)_2_D_3_) from VD_3_ was reviewed by Geng et al. (20). This occurred in the liver and kidney in two steps, mediated by 25-hydroxylase1 and α-hydroxylase enzymes (5).

The common level of VD_3_ in layer diets is about 2,200 IU/kg (21, 22), while the commercial egg breeders' guides recommend 2,500 IU/kg in diets (14), and 3,000–5,000 IU/kg is recommended by commercial producers (18). The Ca-binding protein implicated in the active transport of Ca across the intestinal wall requires VD_3_ (18, 22). In the literature, VD_3_ has many health, immunological, and physiological benefits (5), and it is a vital vitamin that plays a considerable role in the development of muscle, skeletal health, and in sustaining the homeostasis of calcium and phosphorus (18, 23, 24). Formation of eggshell and health of bone in laying hens is essential and involves the integration between the metabolism of Ca, P, and VD_3_ (5, 18, 25). Rodriguez-Lecompte et al. (26) and Manolagas et al. (27) have reported that VD_3_ or the active form of VD_3_, 25-(OH)D, both have strong immunomodulatory properties with a gradually ultimate help of T cells (Th2).

The commercial hybrid laying hens produce eggs at a higher rate due to increasing genetic potential and improvement in farming and nutrition strategies. Consequently, taken together, factors affecting the requirements of Ca and VD_3_ of laying hens need further investigation, particularly during the late phase of production, due to a decline in laying performance, the quality of eggs and shells, and physiological and immunological adaption. Hence, we hypothesized that the Ca and VD_3_ requirements of laying hens increased during late stage of production and increasing Ca and/or VD_3_ may improve production and quality of eggs and profits for laying hens farmers. Thus, this work examines the integration between different concentrations of Ca (3.5, 4.0, and 4.5%) and a (VD_3_) 800-, 1,000-, 1,200-IU/kg diet on the productive traits, blood biochemistry, and immune response of laying hens during the late phase of laying brown eggs.

## Materials and Methods

The scientific committee of the Poultry Production Department, Faculty of Agriculture, Mansoura University, approved the present experiment. The protocol number was DF-715-155-1441 H. The committee recommended that care and handling of the animal maintained rights and welfare and minimized stress (Directive 2010/63/EU).

### Laying Hens and Husbandry

The research was performed at the Poultry Research Unit, Qalabsho Center of Agricultural Researches and Experiments, Faculty of Agriculture, Mansour University, Egypt, from July to September 2018. This is the hottest period of the year in Egypt; the relative humidity and ambient temperature during the experimental period ranged between 54 and 70% and 22.8 and 35.6°C, respectively. The birds were exposed to hot weather conditions, and hens showed symptoms of high ambient temperatures, such as lying down in cages, wing flapping, and panting. Nonetheless, we did not discuss the heat stress in the Results and Discussion section, due to a lack of a control group for heat stress that was kept under optimum temperature (25°C).

360 H&N Brown Nick layers (60-wk-old) were assigned randomly into nine treatments, consisting of five replicates of eight hens. Hens were reared in open-sided laying cages (eight hens/cage with one feeder and two nipples); the size of each cage was 60 × 120 × 50 cm. The hens were submitted to a light program of 16:8 hrs light/dark cycle and received mash feed and tap water *ad libitum*.

### Experimental Diets

Nine experimental diets, consisting of three levels of Ca (3.5, 4.0, and 4.5%) with three levels of supplemented vitamin D_3_ at the dosage of an 800-, 1,000-, 1,200-IU/kg diet or as total of 3,800, 4,000, and 4,200 IC VD_3_ were fed during the experimental period from 60 to 72 wk of age (Table 1). The control diet in this experiment was that contained 3.5% Ca level with 800 IU VD_3_.

**Table 1:** Formulation and proximate analyses of the experimental diets (g/kg) on dry matter basis fed to Brown egg layers strain from 60 to 72 week of age.

**Ingredients (g/kg)**	**Calcium level (g/kg)**		
	**35**	**40**	**45**
Ground yellow corn	628	625.1	620.2
Soybean meal, 44% CP	245	236	215
Corn gluten meal, 60% CP	12.7	19	35
Ground limestone	83	96	109
Dicalcium phosphate	12.0	12.0	12.0
Vitamin and mineral Premix^a^	3.0	3.0	3.0
Sodium chloride	3.0	3.0	3.0
L-Lysine-HCl	0.4	0.5	1.0
DL-Methionine	1.9	1.9	1.8
Sand	11.0	3.5	0.0
Total	1,000	1,000	1,000
**Calculated analysis and determined values (as fed basis), g/kg**			
Metabolizable energy (ME), MJ/kg^2^	11.30	11.30	11.30
Crude protein^b^	171	172	171
Dry matter^b^	902	899	900
Ether extract^b^	31.5	31.8	31.9
Crude fiber^b^	27.1	25.0	22.7
Non-phytate phosphorus^c^	3.43	3.41	3.36
Calcium^c^	35.3	40.2	45.1
Methionine^c^	4.73	4.76	4.76
Methionine + Cystine^c^	7.62	7.65	7.68
Lysine^c^	8.67	8.56	8.54
Ash^b^	101	114	128
Nitrogen-free extract^b^	731	567.4	558.2
Total vitamin D_3_, IU/kg^c^	3,000	3,000	3,000

a *Each 3 kg of premix contained: Vit A, 12 million IU; VD_3_, 1 million IU; Vit. E, 20 g; Vit. K_3_, 3 g; Vit. B_1_, 3 g; Vit. B_2_, 8 g; Vit. B_6_,3 g; Vit. B_12_, 15 mg; Ca pantothenate,12 g; niacin, 40 g; folic acid, 1.5 g; biotin, 50 mg; choline chloride, 600 g; Mn, 80 g; Zn, 75 g; Fe, 40 g; Cu, 10 g; I, 2 g; Se, 0.3 g; Co, 0.25 g; and CaCo_3_ as a carrier*.

b *Determined values*.

c *Calculated analysis*.

The total VD_3_ amounts in the tested diets were 3,800, 4,000, and 4,200 IU/kg. The VD in feedstuffs is found in the form of VD_2_, which has a small activity for poultry (17); thus, it was not considered in the calculation of the total VD_3_ in the diet. The cholecalciferol VD_3_ was of powder feed grade, a product of Polifar Group Limited, Nanjing, Jiangsu, China. The diets were formulated according to NRC (17). The diets met or surpassed the nutrient needs specified in the H&N new management guide (28). The experimental diets were analyzed using the official methods of analysis (29) dry matter, crude protein, ether extract, crude fiber, and ash.

### Laying Performance

Daily records of laying rate (LR), feed intake (DFI), egg weight (EW), egg mass (EM), and mortality rate were recorded and used to calculate the production indexes in a 28-day period as follows:

$$\begin{array}{l} \text{Egg production index = }(\text{Average egg mass/day }\times \text{ percent}\\ \text{survival rate})\text{/Feed conversion rate }\times \text{ 10}. \end{array}$$

Bodyweight change (BWC) was also estimated from the differences between the initial and final body weights during the testing period.

### Egg Quality Measurements

Eggs (30 eggs per treatment) were selected randomly at 72 wk of age to represent equally all replicates and used in the determination of the exterior and interior egg quality characteristics, as cited by Burke and Attia (30) and Attia et al. (31, 32). These characteristics involved EW and its comparative constituents (albumen, yolk, and shell), Haugh units, yolk color score (YCS), yolk index (YI), shell thickness (ST), and egg-shape index (ESI). The eggshell quality was measured at three different points of the shell in the mid-point and at the two ends of eggs, and the values was used for the calculation of the mean value of egg shell thickness. Shell weight per unit surface area (SWUSA) was determined by dividing shell weight by the egg surface area (ESA; cm^2^). These measurements were done as reported by Burke and Attia (30) and Attia et al. (31, 32).

Scanning electronic microscopy (SEM) images of the eggshells were taken according to Stefanello et al. (33) using two samples of the eggshell of each egg collected at 72 wk of age. The number of eggshells was one per replicate of each of the nine treatments. Eggshell free of shell membrane was obtained after breaking open the eggs. The shell membrane was removed by immersion of the samples in a solution of 0.15% sodium hydroxide, 4.12% sodium chloride, and 6% sodium hypochlorite. Tap water was used to wash the shells, which were then air-dried at room temperature (27°C). The images were made using 0.5 cm^2^ of the membrane-free eggshell from each replicate of each treatment using a Shimadzu SS-550 Super Scan instrument (Shimadzu Corporation, EVISA, Kyoto, Japan).

### Blood Parameters

Five milliliter blood samples were collected from wing vein of six hens per group of 72-wk-old in two blood tubes, with and without heparin. The hens used for measuring blood constituents were selected with hard shell eggs in the uterus. Blood samples were centrifugated at 1,716 g for 15 min to separate the plasma and serum, which were kept at 20°C until analysis. Serum total protein, albumin, globulin, triglycerides (Trig.), total cholesterol (TC), and high-density lipoprotein-cholesterol (HDL-C) were determined. Plasma calcium (Ca) and inorganic P (Pi), total antioxidant capacity (TAC), malondialdehyde (MDA), superoxide dismutase (SOD), and catalase (CAT) were measured. Aspartate aminotransferase (AST); Alanine aminotransferase (ALT); alkaline phosphate (AlkP); α-, β-, and γ-globulin; immunoglobulin IgG, IgM, IgA, estrogen, and parathyroid hormone (PTH) were also determined. The measurements were made using commercial diagnostic kits (34) as cited by Attia et al. (35, 36). The antibody titer for avian influenza and Newcastle disease virus were determined using commercial enzyme-linked immunosorbent assay (ELISA) kits as cited by Attia et al. (35, 36).

### Reproductive Inner Organs

Five hens were randomly selected from each treatment and slaughtered according to Islamic methods (35). After complete bleeding, the hens were opened, and the ovary and reproductive tract were dissected. Then the weight of the ovary and oviduct parts (infundibulum, magnum, isthmus, and uterus) were recorded, and their proportions relative to the live bodyweight of the hens were estimated.

### Statistical Analysis

The analytical processing of results was performed by using a two-way analysis of variance (Ca, VD_3_, and their interaction) of the GLM procedure of the Statistical Analysis System (37) using the replicate as the experimental unit. Data were transformed to arcsine to normalize the distribution. Differences between means were tested using the Student–Newman–Keuls test at *P* < 0.05 (37). The *P*-values between 0.10 and <0.05 were considered as a trend.

## Results and Discussion

### Laying Hens Performance

Table 2 shows the influence of Ca and/or VD_3_ level on the production traits of laying hens during the late phase. Initial BW of laying hens was not different between the experimental groups, indicating a random distribution of hens; thus, data were not presented. The results suggest that Ca and/or VD_3_ levels had a significant effect on most laying performance traits, except for feed intake. EW was gradually increased with increasing Ca levels. The interaction effect indicates that increasing Ca level from 3.5 to 4 or 4.5% similarly improved LR, EM, FCR, EPI, and BWG. Increasing the Ca level from 3.5 to 4% did not influence EW, but a further increase to 4.5% increased EW; thus, the difference between 3.5 and 4.5% Ca groups was significant. These results reveal that 4% Ca was adequate for the performance of layers of 60–72 wk of age and confirmed the suggested Ca requirements (4.1% during 45–70 wk of age) in the H&N management guide (28) and those by Rao and Roland (38) and Zhang and Coon (39). These may differ due to the role of Ca in regulating the reproductive hormones and ovary growth (5). Nascimento et al. (40) found that there was no interaction between the different sources of vitamin D at a 2,000-IU/kg diet from VD_3_, 25-(OH)D_3_, 1,25-(OH)_2_D_3_, and four calcium levels from 2.85 to 5.25%, with intervals of 0.80% in all egg production traits. However, a significant quadratic component of the contrast analysis for the level of calcium in LR and FCR was observed, showing better productive characteristics at 4.12 and 4.09% Ca, respectively. In contrast, the level of Ca did not affect the EW. Nonetheless, the source of VD_3_ influenced (*P* < 0.05) LR, FCR, and EW, showing improved results of laying hens with 25-(OH)D_3_ and cholecalciferol (40).

**Table 2:** Productive performance of brown egg layers fed three levels of Ca, supplemented with three levels of VD_3_ from 60 to 72 wk of age.

**Treatments**	**Productive performance**						
	**Laying rate,%**	**Egg weight, g**	**Egg mass, g/day**	**Feed intake, g/day**	**Feed conversion ratio, kg/kg**	**Egg production index**	**Bodyweight gain, g**
**Calcium level**							
3.5%	72.6^c^	74.2^c^	54.0^b^	104	1.93^b^	28.4^b^	608^b^
4.0%	76.3^a^	75.1^b^	57.4^a^	104	1.81^a^	31.9^a^	710^a^
4.5%	74.1^b^	76.4^a^	56.7^a^	104	1.81^a^	31.2 ^a^	721^a^
**VD**_**3**_ **level**							
800 IU	70.6^b^	73.3^b^	51.9^b^	103	1.99^c^	26.4^c^	576^b^
1000 IU	76.6^a^	76.5^a^	58.7^a^	104	1.77^a^	32.3^a^	748^a^
1200 IU	75.7^a^	75.9^a^	57.6^a^	105	1.82^b^	31.8^b^	715^a^
**Interaction Ca × VD**_**3**_ **effect**							
3.5 800	66.4^c^	71.4	47.5^c^	102	2.14^a^	22.2^e^	375^b^
3.5 1,000	77.1^a^^b^	75.8	58.5^a^^b^	105	1.79^d^	32.8^a^^b^	721^a^
3.5 1,200	74.2^a^^b^	75.4	56.1^a^^b^	105	1.87^b^^c^^d^	30.3^b^^c^^d^	727^a^
4.0 800	72.8^b^	73.5	53.5^b^	103	1.93^b^	27.8^d^	704^a^
4.0 1,000	78.0^a^	75.9	59.3^a^	104	1.75^e^	34.1^a^	775^a^
4.0 1,200	78.0^a^	75.8	59.3^a^	105	1.77^d^^e^	33.7^a^	685^a^
4.5 800	72.7^b^	75.2	54.6^a^^b^	104	1.89^b^^c^	29.1^c^^d^	650^a^
4.5 1,000	74.9^a^^b^	77.7	58.2^a^^b^	103	1.78^d^^e^	32.9^a^^b^	747^a^
4.5 1,200	74.8^a^^b^	76.5	57.3^a^^b^	104	1.82^c^^d^^e^	31.6^a^^b^^c^	737^a^
**Statistical analyses**							
SEM	1.14	0.752	1.24	2.67	0.029	1.51	39.2
***P*****-values**							
Ca	0.001	0.001	0.001	0.953	0.001	0.001	0.001
VD_3_	0.001	0.001	0.001	0.094	0.001	0.001	0.001
Interaction	0.001	0.079	0.001	0.499	0.001	0.002	0.002

a,b,c,d,e *Means in the same column under the same effect with different superscripts are significantly different (P < 0.05). SEM, standard error of the means*.

It was observed that of VD_3_ at a 1,000- and 1,200-IU/kg diet to 3.5 and 4% Ca diets significantly increased LR, EM, and improved FCR. In addition, VD_3_ supplementation at a 1,000- and 1,200-IU/kg diet to 3.5% Ca markedly increased the BWG of laying hens (Table 2). Increasing VD_3_ to 1,000 IU significantly increased EW, and the effect was saturated at this level. The addition of VD_3_ at 1,000 and 1,200 IU to different Ca levels increased the EPI of hens on different Ca levels except for hens fed 4.5% Ca supplemented with a 1,200-IU/kg diet. These results reveal that supplementation with 1,000 IU of VD_3_/kg diet or a total of 4,000 IU/kg is adequate for egg production traits of hens fed 3.5% Ca level.

The present observations indicate that VD_3_ content in the diet containing 3.5% with a total of VD_3_ at 3,000 IU/kg (Table 1) was not adequate for H&N Brown laying hens in the late phase. It should be mentioned that the H&N Brown nutrition management guide recommends 2,500 IU for laying during the laying period with no specific recommendations and/or considerations for each stage of laying. In addition, 3,000–5,000 IU of VD_3_ for laying hens during different stages of production was recommended (18). It is evident that as the metabolism of nutrients decreases with age (5, 41), the VD_3_ requirements should be reinvestigated in relation to stage of laying considering the impact of aging on VD_3_ requirements and due to VD_3_ and its metabolites playing a vital role in the uptake, deposition, and excretion of Ca (5, 21, 42). Likewise, other authors (5, 24, 27) showed similar findings. The VD_3_ is essential for the formation of Ca-bound proteins, which are involved in the active transport of Ca across the intestine (28), and in lipoprotein, the precursor of yolk formation (5, 20, 21).

### Egg Quality Traits

The results indicate that Ca and the interaction between Ca and VD_3_ levels has significant effects on egg quality traits (Table 3).

**Table 3:** Exterior and interior egg quality traits of brown Egg layers fed three levels of Ca supplemented with three levels of VD_3_ from 60 to 72 wk of age.

**Treatments**	**Yolk, %**	**Albumen, %**	**Y: A ratio**	**Haugh unit score**	**Yolk index**	**Yolk color**	**Shape index**	**Shell, %**	**Shell thickness, μm**	**SWUSA, mg/cm^**2**^**
**Calcium level**										
3.5%	27.5^b^	63.6^a^	0.433^b^	75.7^c^	35.2^a^	7.53^b^	84.5^a^	8.92^c^	426^b^	67.1^c^
4.0%	30.8^a^	58.8^b^	0.525^a^	81.3^b^	31.0^b^	7.96^a^	84.3^a^	10.4^b^	467^a^	72.3^b^
4.5%	30.9^a^	58.3^c^	0.531^a^	82.6^a^	31.1^b^	8.00^a^	83.3^b^	10.7^a^	466^a^	75.0^a^
**VD**_**3**_ **level**										
800 IU	29.8	60.5	0.495	78.4^b^	32.1	7.78	84.5^a^	9.78^c^	430^b^	69.9^c^
1,000 IU	29.8	60.1	0.498	80.6^a^	32.7	7.87	83.6^b^	10.1^b^	466^a^	71.4^b^
1,200 IU	29.7	60.1	0.456	80.5^a^	32.4	7.84	84.0^a^^b^	10.3^a^	463^a^	73.1^a^
**Interaction Ca × VD**_**3**_ **effect**										
3.5 800	27.4^d^	64.1^a^	0.429^d^	70.5^d^	34.0^b^	7.47^b^	84.4^a^^b^	8.47^d^	355^d^	62.4^e^
3.5 1,000	27.7^d^	63.3^a^	0.439^d^	77.3^c^	35.6^a^	7.67^b^	83.9^a^^b^^c^	9.02^c^	467^a^^b^^c^	67.7^d^
3.5 1,200	27.4^d^	63.4^a^	0.432^d^	79.3^b^	35.8^a^	7.47^b^	85.3^a^	9.28^c^	456^c^	71.2^c^
4.0 800	31.3^a^^b^	58.2^c^^d^	0.537^a^^b^	82.1^a^	31.3^c^	8.00^a^^b^	84.8^a^^b^	10.5^b^	475^a^	73.3^b^
4.0 1,000	30.2^c^	59.4^b^	0.508^c^	82.1^a^	31.4^c^	7.53^a^^b^	84.3^a^^b^	10.4^b^	454^c^	71.2^c^
4.0 1,200	30.9^a^^b^^c^	58.6^b^^c^^d^	0.529^b^	79.7^b^	30.4^c^	8.33^a^	83.8^a^^b^^c^	10.4^b^	471^a^^b^	72.4^b^^c^
4.5 800	30.6^b^^c^	59.0^b^^c^	0.518^b^^c^	82.7^a^	31.1^c^	7.87^b^	84.3^a^^b^^c^	10.4^b^	469^a^^b^^c^	73.9^a^^b^
4.5 1,000	31.6^a^	57.7^d^	0.548^a^	82.6^a^	31.1^c^	8.40^a^	82.5^c^	10.8^a^^b^	476^a^	75.4^a^
4.5 1,200	30.6^b^^c^	58.3^c^^d^	0.526^b^	82.6^a^	31.1^c^	7.73^b^	83.1^b^^c^	11.1^a^	463^a^^b^^c^	75.7^a^
**Statistical variance**										
SEM	0.219	0.264	0.005	0.682	0.407	0.138	0.422	0.120	3.92	0.527
***P*****-values**										
Ca	0.001	0.001	0.001	0.001	0.001	0.001	0.001	0.001	0.001	0.001
VD_3_	0.645	0.162	0.737	0.001	0.240	0.717	0.036	0.001	0.001	0.001
Interaction	0.001	0.001	0.001	0.001	0.021	0.001	0.040	0.002	0.001	0.001

a,b,c,d,e *Means in the same column under the same effect with different superscripts are significantly different (P < 0.05). SEM, standard error of the means; Y, A ratio; yolk, albumen ratio; SWUSA, shell weight per unit surface area*.

Substantial effects of VD_3_ on only the Haugh unit score, shape index, and shell quality traits were recorded. Similarly, VD sources such as VD_3_, and 25-(OH) D_3_ at 2,000 IU increased the Haugh unit score, following Nascimento et al. (40).

The interaction between Ca level and VD_3_ reveals that increasing the Ca level from 3.5 to 4 or 4.5% markedly and similarly increased the percentage of yolk, yolk:albumen ratio, Haugh unit score, shell percentage, ST, and SWUSA. The results indicated that 4% Ca was adequate for improving egg quality characteristics during the late stage of egg production and increasing the Ca level to 4.5% had no positive effects. The improvement in egg and shell quality traits of hens fed 4% Ca suggests an increased Ca availability for eggshell formation, in accordance with other researchers in this field (16, 40). This may be due to the role of Ca in regulating the reproductive hormones and ovary growth and its vital role in the eggshell formation and as a critical agent for preserving egg quality (5, 16, 21). It was cited that every increase in the quantity of available Ca may improve shell quality and bone-breaking strength, with the effect being linear (40, 43).

The supplementation with VD_3_ at 1,000 IU to 4% Ca diet substantially reduced the yolk percentage of hens on 4% Ca, compared to the same Ca group with 800 IU of VD_3_, and yolk:albumen ratio, compared to 800 and 1,200 IU. However, 1,000 IU of VD_3_ increased percentage of yolk, yolk:albumen ratio, and yolk color of those fed 4.5% Ca compared to the other groups at the same Ca level. The yolk index was significantly increased due to supplementation of 3.5% Ca with 1,000 and 1,200 IU VD_3_ compared to 800 IU added to the same level of Ca.

Albumen percentage showed a marked increase due to VD_3_ supplementation at 1,000 IU to 4.0% Ca, compared to the 800 IU of VD_3_ supplemented to the same level of Ca. On the other hand, 1,000 IU of VD_3_ significantly decreased albumen percentage of hens fed 4.5% Ca, compared to 800 IU of VD_3_ added to the same level of Ca. These changes are contrary to the changes showed in yolk percentage.

Haugh unit, the mirror of albumen quality, showed a considerable increase stepwise, with increasing VD_3_ addition to 3.5% Ca diet, but 1,200 IU of VD_3_ to 4% Ca diet significantly decreasing Haugh unit, compared to the other levels of VD_3_ supplemented to the same level of Ca. The effect of VD_3_ is dependent on concentrations of Ca level. The highest egg shape index was from hens fed 3.5% Ca level supplemented with 1,200 IU while the smallest was from hens fed 4.5% Ca supplemented with 1,000 IU of VD_3_. The fortification with VD_3_ at 1,000 and 1,200 IU to 3.5% Ca feed substantially increased the percentage of the shell and shell thickness similarly compared to those on 800 IU of VD_3_. The increase in the SWUSA was linear, maybe due to the correction for egg surface area (31, 32).

The effect of VD_3_ on eggshell quality of hens fed adequate Ca levels (4 and 4.5%) was less pronounced and depended on the type of measurements. These results demonstrated that the impact of VD_3_ on eggshell quality depends on dietary Ca level being more pronounced at inadequate Ca intake, according to other research (5, 21, 27). VD metabolites are essential for Ca-binding protein that involved in the active transport of Ca across the intestinal wall, which is essential for eggshell formation (5, 21, 23). The formation of Ca-binding proteins in different tissues (intestine, kidney, and uterus) required VD metabolites at both stages of transcriptional and post-transcriptional. Calcium-binding proteins enhance the absorption of calcium in the gut, recovery from the urine, and shell deposition (17, 20).

**Figure 1:**
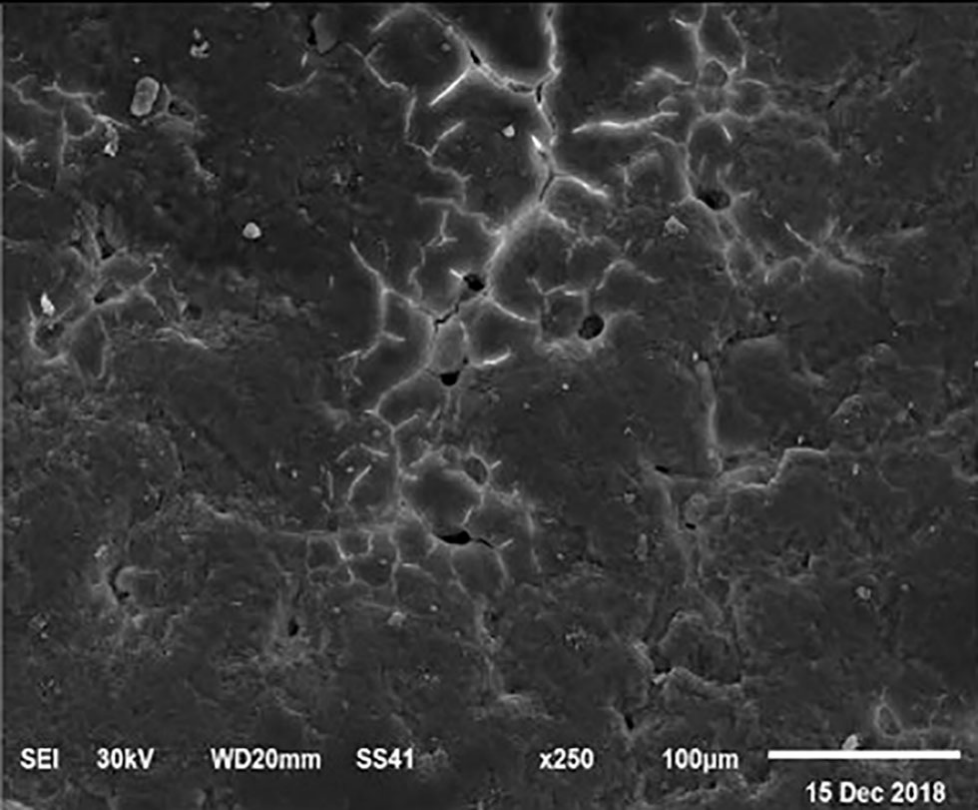
SEM photo of the eggshell of laying hens fed the control diet of 3.5% Ca with 800-IU VD_3_/kg diet_._ The figure shows a low density of mammillary buttons, which results in a reduction of strength, according to the significantly lowest eggshell thickness and SWUSA (*P* < 0.05) recorded in this experimental group. It is important to underline the great relative interstitial area between mammillary formations, which makes the egg more susceptible to breaking along these cracks.

**Figure 2:**
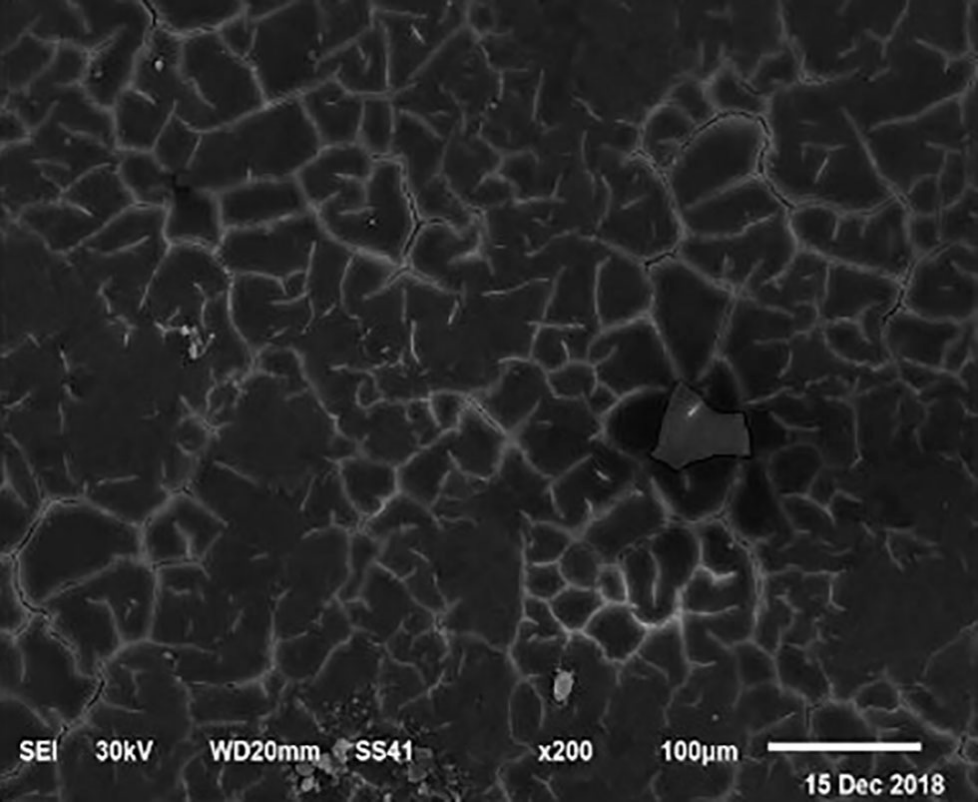
SEM photo of the eggshell of laying hens fed a diet containing 4.0% Ca with 800-IU VD_3_/kg diet. Compared to Figure 1, a slight increase in the number of mammillary buttons associated with an increase of the larger size can be detected.

**Figure 3:**
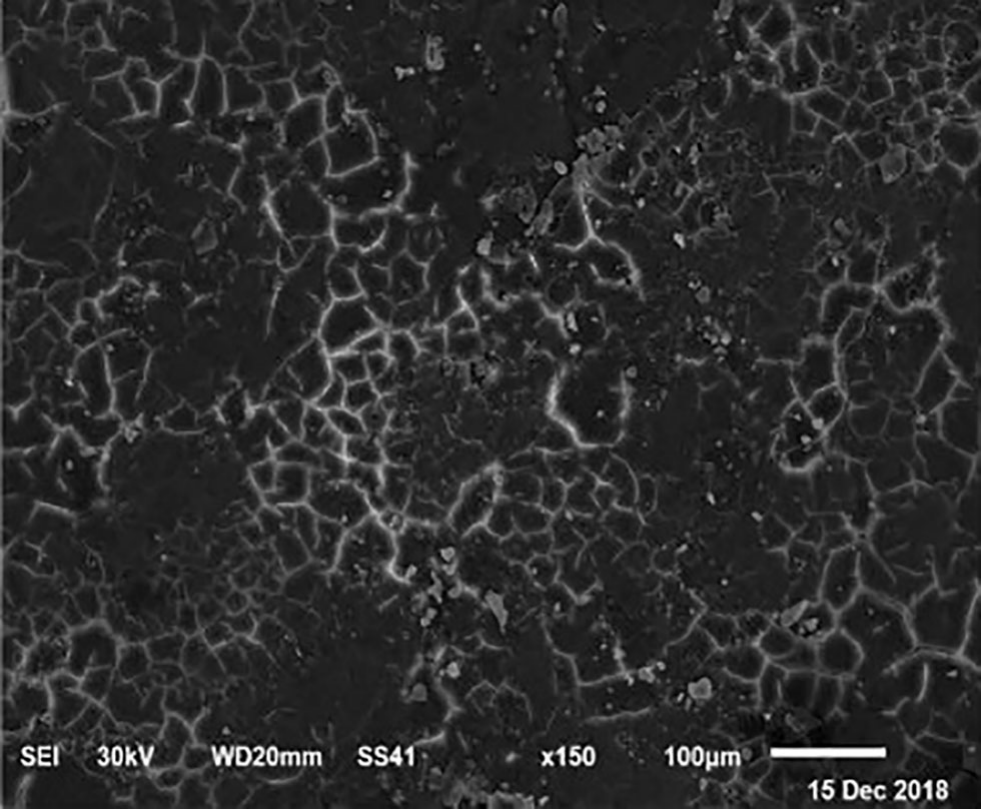
SEM photo of the eggshell of laying hens fed a diet containing 4.5% Ca with 800-IU VD_3_/kg diet. It is possible to observe an increase in the number of mammillary buttons, compared to Figure 1, by increasing only the levels of Ca.

**Figure 4:**
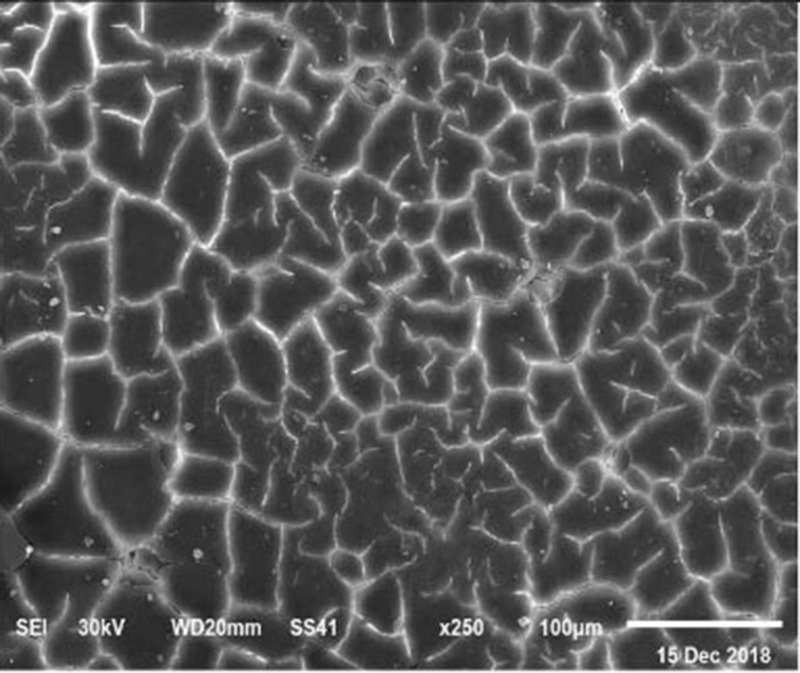
SEM photo of the eggshell of laying hens fed a diet containing 3.5% Ca with 1,000-IU VD_3_/kg diet_._ This figure shows a similar mammillary buttons distribution, but the interstitial area seems less evident than in Figure 1.

**Figure 5:**
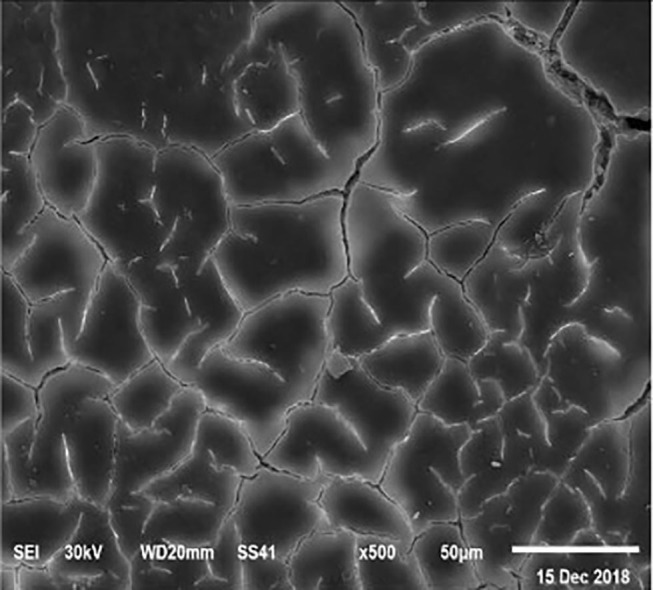
SEM photo of the eggshell of laying hens fed a diet containing 4.0% Ca with 1,000-IU VD_3_/kg diet_._ In this figure, it is possible to observe a greater relative interstitial area between mammillary formations that negatively influences the strength of the eggshell. The higher interstitial area is an indicator of the degree of elasticity of the eggshell.

**Figure 6:**
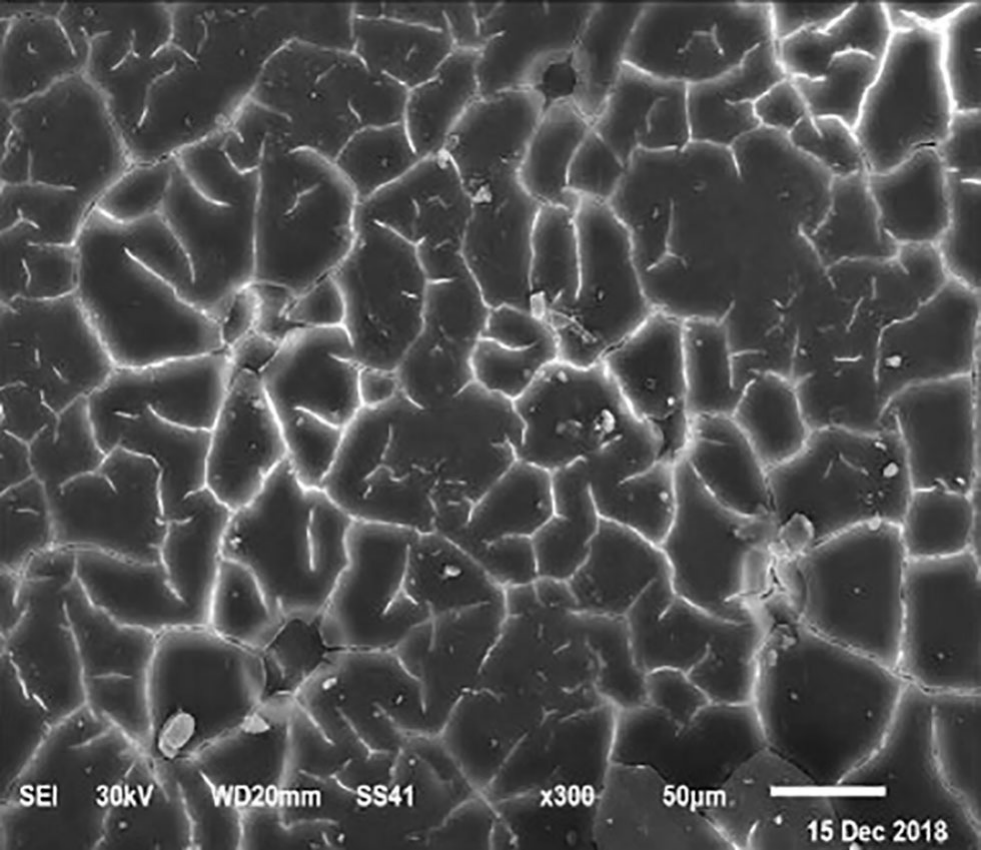
SEM photo of the eggshell of laying hens fed a diet containing 4.5% Ca with 1,000-IU VD_3_/kg diet_._ Compared to Figure 5, a reduction of the interstitial area between mammillary buttons was observed, but no differences were detected concerning the mammillary buttons density.

**Figure 7:**
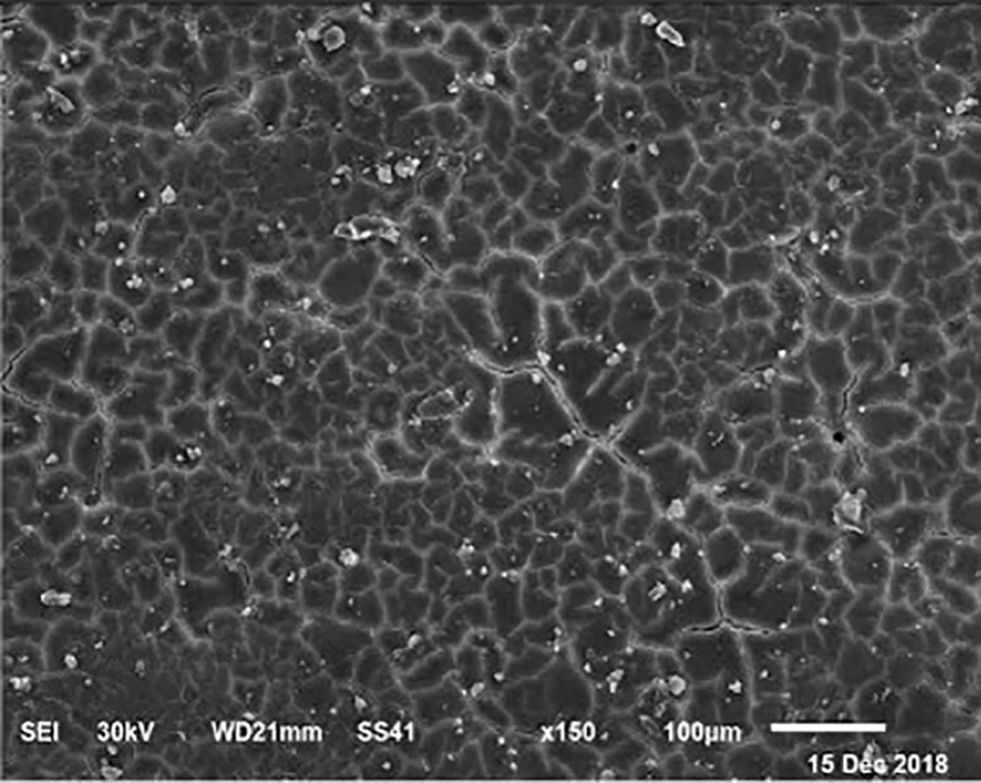
SEM photo of the eggshell of laying hens fed a diet containing 3.5% Ca with 1,200-IU VD_3_/kg diet_._ In this figure, it is possible to observe that rather than Ca percentage, VD_3_ is responsible for the size of mammillary buttons.

**Figure 8:**
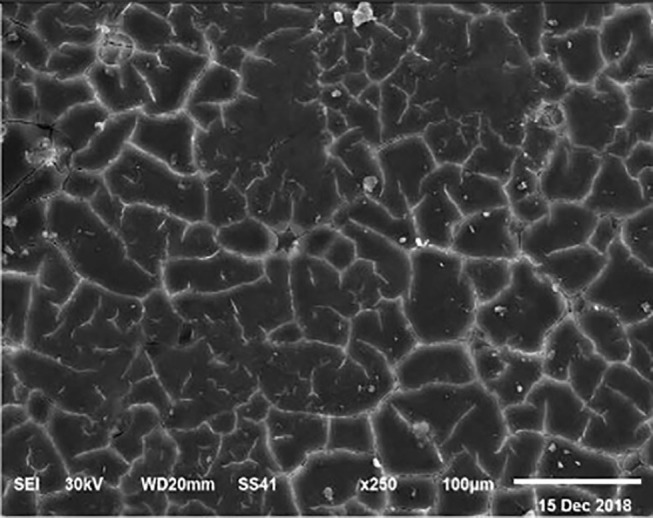
SEM photo of the eggshell of laying hens fed a diet containing 4.0% Ca with 1,200 IU-VD_3_/kg diet_._ In this figure, it is possible to observe the highest density and percentage of mammillary buttons in the eggs from laying hens fed the diet with the 4.0 and 4.5% Ca and 1,200 IU VD_3_, according to the highest (*P* < 0.05) shell thickness and SWUSA.

**Figure 9:**
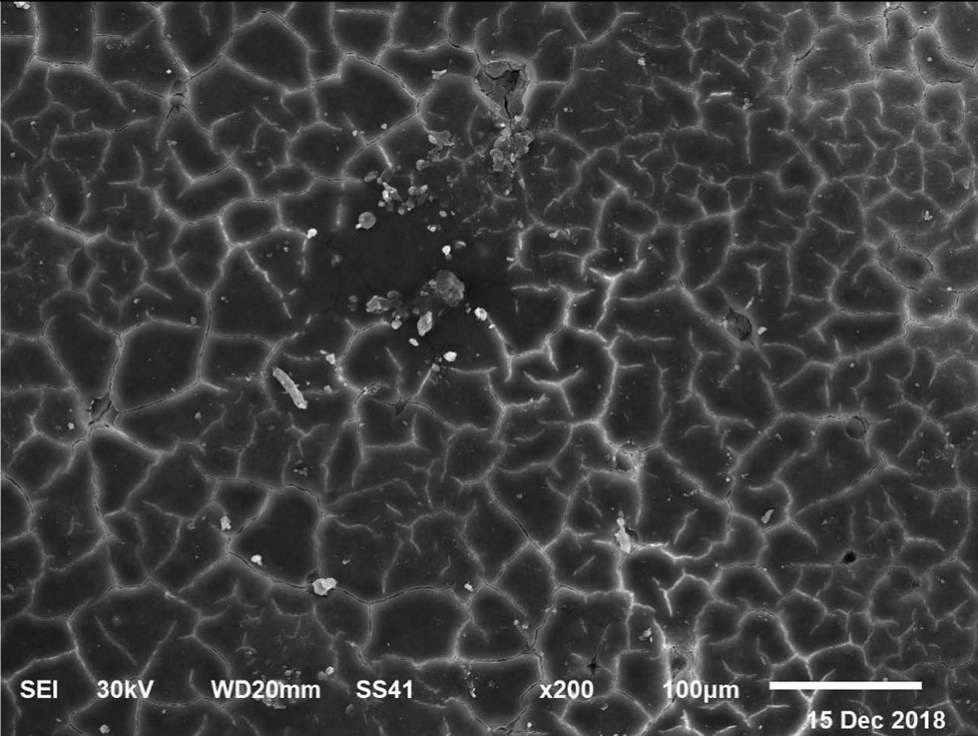
SEM photo of the eggshell of laying hens fed a diet containing 4.5% Ca with 1,200-IU VD_3_/kg diet_._ In this figure, it is possible to observe the highest density and percentage of mammillary buttons in the eggs from laying hens fed the diet with the 4.0 and 4.5% Ca and 1,200 IU VD_3_, according to the highest (*P* < 0.05) shell thickness and SWUSA.

The electronic microscope images (Figures 1–9) showed an increase in ultra-structure of eggshell due to increasing Ca level and VD_3_ supplementation, as manifested by the different distribution of mammillary buttons, which became larger as the dietary levels of Ca and VD_3_ increased (Figures 2–9). The eggshell, evaluated by electronic microscopy, consists of several morphologically different regions and also contains thousands of gas exchange pores. The outermost layer is the cuticle that acts as a physical barrier to water and bacterial contamination. The specific nucleation sites on the outer surface of the outer shell membrane attract calcium salts for the formation of the calcified layers (cone or mammillary layer), which may be influenced by the enzymatic activity, and trace minerals act as cofactors of such a process. Each palisade column grows from one mammillary button, and as the calcification mechanism proceeds, they provide greater resistance to the shell (44).

The increased strength of shells, as well as the reduction in the egg loss, is an important goal that has economic importance in commercial terms. The quality of the eggshell has been considered to be affected by organic components particularly protein (9, 10) and the inorganic components (45). Therefore, the palisade layer comprises approximately two-thirds of the entire thickness of the shell (46). In this regard, the palisade layer showed a linear reduction in the number of mammillary buttons in the eggshell of hens fed, increasing Mn, Zn, and Cu levels (33). As reported by Stefanello et al. (33), supplementation with organic trace elements can exert an influence on the formation of the palisade layer, resulting in larger mammillary buttons. Our results showed that the group fed a diet containing 3.5% Ca supplemented with 800 IU VD_3_ had a clutter on the distribution of mammillary buttons on the inner surface of the shell (Figure 1). From these results, we can speculate that the palisade layer and the number of mammillary buttons present in the shell may also influence the quality of the shell.

### Plasma and Tibia Minerals Content

Table 4 shows the influence of dietary Ca and VD_3_ on plasma minerals and tibia characteristics. Dietary Ca and/or VD_3_ levels did not affect plasma Ca, Pi, and Ca:Pi levels.

**Table 4:** Blood plasma minerals and tibia characteristics of 72-wk-old brown egg layers fed three levels of Ca supplemented with three levels of VD_3_ from 60 to 72 wk of age.

**Treatments**	**Ca, mq/L**	**Pi, mg/dL**	**Ca: Pi Ratio**	**Tibia ash, %**	**Tibia Ca, %**	**Tibia P, %**	**Tibia Ca:P ratio**
**Calcium level**							
3.5% Ca	27.3	6.61	4.13	62.5^c^	30.6^b^	12.8^b^	2.41^a^
4.0% Ca	27.9	6.51	4.28	64.0^b^	32.7^a^	14.6^a^	2.25^b^
4.5% Ca	28.0	6.39	4.41	64.6^a^	32.7^a^	14.7^a^	2.23^b^
**VD**_**3**_ **level**							
800 IU	27.6	6.54	4.21	63.1^c^	31.3^b^	13.5^b^	2.34
1,000 IU	27.6	6.66	4.16	63.8^b^	32.2^a^	14.2^a^	2.25
1,200 IU	27.9	6.30	4.44	64.2^a^	32.6^a^	14.3^a^	2.30
**Interaction Ca × VD**_**3**_ **effect**							
3.5 800	26.3	6.24	4.21	60.9^c^	28.7^c^	11.5^c^	2.50
3.5 1,000	27.5	7.02	3.92	62.7^b^	31.0^b^	13.3^b^	2.33
3.5 1,200	28.0	6.56	4.27	63.9^a^	32.2^a^	13.5^b^	2.39
4.0 800	27.9	6.55	4.26	64.0^a^	32.8^a^	14.5^a^^b^	2.26
4.0 1,000	27.6	6.68	4.13	63.9^a^	33.0^a^	14.5^a^^b^	2.28
4.0 1,200	28.1	6.32	4.45	64.1^a^	32.3^a^	14.7^a^^b^	2.21
4.5 800	28.5	6.85	4.16	64.4^a^	32.3^a^	14.5^a^^b^	2.24
4.5 1,000	27.8	6.28	4.43	64.6^a^	32.7^a^	15.2^a^	2.15
4.5 1,200	27.8	6.03	4.61	64.6^a^	33.1^a^	14.4^a^^b^	2.30
**Statistical analyses**							
SEM	0.905	0.317	0.182	0.267	0.345	0.353	0.064
***P*****-values**							
Ca	0.544	0.706	0.147	0.001	0.001	0.001	0.003
VD_3_	0.852	0.386	0.082	0.001	0.001	0.015	0.304
Interaction	0.688	0.288	0.407	0.001	0.001	0.030	0.353

a,b,c *Means in the same column under the same effect with different superscripts are significantly different (P < 0.05). SEM, standard error of the means; Ca, calcium; Pi, inorganic phosphorus; Ca:Pi, calcium to inorganic phosphorus ratio*.

This indicates that Ca and VD_3_ in the control diets (3.5% Ca and 800 IU of supplemented VD_3_ or as a total VD_3_ at a 3,800-IU/kg diet) were adequate to maintain plasma Ca and Pi concentation at normal levels. Also, Nascimento et al. (20, 40) demonstrated that there were no effects of VD sources at 2,000 IU from VD_3_, 25- (OH) D3, and 1, 25 (OH)2 D3 on plasma Ca. Also, Susanna et al. (47) observed that dietary VD_3_ at 3,000 IU from a single source or at 1,500 IU of each VD_3_ and 25 (OH) D3 did not affect plasma Ca level, but decreased plasma Pi of 34-wk-old laying hens. This may be due to hormonal control of Ca and Pi via parathyroid and calcitonin (21, 41, 48).

It was found that ash, Ca, and Pi were affected significantly by dietary Ca and VD_3_ levels, but the impact interfered with the interaction between the two variables. Also, tibia Ca: Pi was affected only by nutritional Ca levels, showing that the Ca:Pi ratio was significantly similarly decreased due to Ca increasing above 3.5%. Increasing Ca level from 3.5 to 4 and 4.5% within the unsupplemented diets significantly and similarly increased tibia ash, Ca, and Pi, showing response saturation at 4% Ca. This suggests that 4% Ca was adequate for bone calcification (5, 37–41). The supplementation of the 3.5% Ca level with VD_3_ at 1,000 and 1,200 IU significantly increased tibia ash and Ca in a stepwise manner and similarly increased Pi. This indicates that supplementation with VD_3_ is beneficial for laying hens from 60 wk of age on for bone calcification (16, 20). These may be due to the role of VD_3_ and its metabolites in the bone matrix (20, 41). It was observed that increasing the VD_3_ supplementation to 1,000 and 1,200 IU within 4 and 4.5% Ca levels did not affect tibia ash and Ca and Pi contents. These results indicate that the response to VD_3_ depends on the dietary Ca levels (5, 14).

### Serum Lipid Metabolites

The effect of different dietary Ca and/or VD_3_ levels on most of the blood serum lipid metabolites was not significant, except for triglycerides, vLDL, and triglyceride:HDL ratio and approached significant for HDL (Table 5).

**Table 5:** Blood serum lipid metabolites of brown egg layers fed three levels of Ca supplemented with three levels of VD_3_ from 60 to 72 wk of age.

**Treatments**	**Cho, mg/dL**	**Trig, mg/dL**	**HDL, mg/dL**	**vLDL, mg/dL**	**LDL, mg/dL**	**Trig: HDL Ratio**	**LDL: HDL Ratio**	**Cholesterol risk factor**	**Cholesterol lowering factor**
**Calcium level**									
3.5% Ca	183	150^a^	57.6	29.9^a^	95.7	2.62^a^	1.68	1.92	3.21
4.0% Ca	180	140^b^	57.6	26.6^b^	95.4	2.32^b^	1.67	1.89	3.14
4.5% Ca	185	133^a^^b^	62.4	27.9^a^^b^	95.3	2.24^b^	1.54	1.95	2.99
**VD**_**3**_ **level**									
800 IU	182	142	58.5	28.3	95.5	2.43	1.64	1.91	3.13
1,000 IU	183	142	59.7	28.4	94.9	2.39	1.61	1.93	3.09
1,200 IU	183	139	58.4	27.8	95.9	2.35	1.64	1.91	3.11
**Interaction Ca × VD**_**3**_ **effect**									
3.5 800	182	149	55.6	29.7	97.1	2.69	1.76	1.89	3.30
3.5 1,000	183	154	57.9	30.7	94.4	2.68	1.64	1.94	3.17
3.5 1,200	184	147	59.3	29.4	95.7	2.49	1.64	1.93	3.14
4.0 800	182	138	58.7	27.5	95.3	2.34	1.62	1.91	3.09
4.0 1,000	179	133	56.8	26.5	95.8	2.34	1.71	1.88	3.18
4.0 1,200	178	129	57.3	25.9	95.1	2.27	1.68	1.88	3.14
4.5 800	183	139	61.2	27.7	94.1	2.26	1.55	1.94	2.99
4.5 1,000	187	140	64.5	27.9	94.8	2.17	1.49	1.98	2.93
4.5 1,200	187	141	61.6	28.2	96.9	2.29	1.58	1.92	3.04
**Statistical variance**									
SEM	4.485	5.974	2.711	1.195	3.258	0.091	0.101	0.053	0.111
***P*****-values**									
Ca	0.253	0.007	0.058	0.007	0.986	0.001	0.184	0.372	0.069
VD_3_	0.971	0.844	0.857	0.844	0.941	0.550	0.931	0.867	0.911
Interaction	0.924	0.847	0.778	0.847	0.954	0.544	0.811	0.905	0.771

a,b *Means in the same column under the same effect with different superscripts significantly different (P < 0.05). SEM. standard error of the means; Cho, cholesterol; Trig, triglycerides; HDL, high density lipoprotein; vLDL, very low density lipoprotein; LDL, low density lipoprotein; Trig:HDL ratio, triglycerlide:high density lipoprotein ratio, LDL:HDL ratio, low density lipoprotein:high density lipoprotein ratio*.

The results demonstrated that Ca levels at 4% considerably decreased triglycerides and vLDL compared to 3.5% Ca. Besides, 4 and 4.5% Ca concentrations substantially decreased serum triglyceride:HDL, compared to 3.5% Ca. Also, this associated with an increase in HDL with increasing Ca level. These decreases could be attributed to the increase in the daily egg mass output of hens fed 4 and 4.5% Ca, due to the use lipoproteins for yolk formation. Lipoproteins, particularly very low density lipoprotein yolk (VLDLy) were formatted under the influence of estrogen. The yolk precursor is synthesized in the liver and transported to the ovary for yolk formation, which would be increased with increasing LR (1, 26, 49).

### Serum Protein Fractions and Liver Index Enzymes

Table 6 displays the impact of different Ca and VD_3_ levels on serum protein fractions and liver indices for leakage enzymes. There were no effects of different Ca levels on serum albumin, AST, ALT, and alkaline phosphatase. The increase in Ca level induced a similar increase in the total serum protein, globulin (specific immune protein) and α-2-globulin compared to 3.5% Ca, but similarly decreased the serum albumin (non-specific immune agent)/globulin ratio and the AST:ALT ratio. These results indicate that Ca is an essential element for the immunity of laying hens, as manifested by the increase in serum total protein and globulin (50–52). Ca deficiency in laying hens can cause bone diseases such as cage layer fatigue and osteoporosis (1, 5, 18, 26).

**Table 6:** Blood serum protein fractions and indices of liver leakage enzymes of brown egg layers fed three levels of Ca, supplemented with three levels of VD_3_ from 60 to 72 wk of age.

**Treatments**	**Total protein, g/dL**	**Albumin, g/dL**	**Globulin, g/dL**	**Albumin: Globulin ratio**	**α1- Glob, %**	**α2- Glob, %**	**β- Glob, %**	**γ- Glob, %**	**AST, U/dL**	**ALT, U/dL**	**AST/ALT ratio**	**AlkP, U/dl**
**Calcium level**												
3.5% Ca	5.51^b^	2.94	2.57^b^	1.15^a^	5.74	4.23^b^	6.40	42.2	85.2	25.1	0.30^a^	82.7
4.0% Ca	6.04^a^	2.84	3.19^a^	0.89^b^	5.36	5.32^a^	7.58	38.5	88.2	23.6	0.27^b^	85.2
4.5% Ca	6.13^a^	2.94	3.18^a^	0.92^b^	5.60	6.23^a^	6.62	39.4	87.9	22.3	0.25^b^	84.5
**VD**_**3**_ **level**												
800 IU	5.50^b^	2.73^b^	2.78^b^	0.99	5.20	5.03	5.89	41.7	85.9^b^	22.9	0.27	86.6
1,000 IU	6.15^a^	3.09^a^	2.05^a^	1.03	5.82	5.41	7.52	40.8	85.3^b^	23.8	0.28	82.2
1,200 IU	6.03^a^	2.89^a^^b^	3.13^a^	0.94	5.68	5.33	7.19	37.6	90.1^a^	24.1	0.27	83.7
**Interaction Ca × VD**_**3**_ **effect**												
3.5 800	4.91	2.59	2.31	1.13	4.58	3.46	5.57^a^^b^	43.3^a^^b^	86.8	25.7	0.30	85.5
3.5 1,000	5.83	3.16	2.66	1.19	5.06	4.53	9.03^a^	43.0^a^^b^	78.7	24.7	0.32	81.0
3.5 1,200	5.79	3.06	2.74	1.12	7.59	4.68	4.62^a^^b^	40.4^a^^b^^c^	90.0	24.9	0.28	81.7
4.0 800	5.77	2.84	2.93	0.97	7.02	5.21	8.10^a^^b^	37.3^b^^c^	85.5	21.8	0.25	87.9
4.0 1,000	6.25	3.01	3.24	0.93	5.42	5.83	5.29^b^	43.2^a^^b^	89.4	23.6	0.26	81.2
4.0 1,200	6.09	2.68	3.42	0.79	3.64	4.92	9.34^a^	34.9^c^	89.8	24.9	0.28	86.6
4.5 800	5.83	2.75	3.08	0.89	4.01	6.43	3.99^b^	44.5^a^	85.4	21.3	0.25	86.3
4.5 1,000	6.36	3.12	3.24	0.97	6.98	5.88	8.26^a^^b^	36.1^c^	87.8	23.1	0.26	84.4
4.5 1,200	6.19	2.94	3.24	0.90	5.81	6.39	7.60^a^^b^	37.6^b^^c^	90.3	22.5	0.25	82.7
**Statistical variance**												
SEM	0.197	0.151	0.103	0.057	1.138	0.655	0.992	1.54	2.219	1.628	0.016	3.075
***P*****-values**												
Ca	0.0005	0.647	0.001	0.001	0.918	0.003	0.316	0.014	0.213	0.126	0.0126	0.592
VD_3_	0.0004	0.012	0.0004	0.125	0.785	0.761	0.117	0.007	0.027	0.662	0.501	0.214
Interaction	0.538	0.333	0.562	0.471	0.056	0.571	0.001	0.001	0.058	0.756	0.562	0.827

a,b,c *Means in the same column under the same effect with different superscripts are significantly different (P < 0.05). SEM, standard error of the means; α1-globulin, alpha 1-globulin; α2-globulin, alpha 2 globulin; β-globulin, beta-globulin; γ-globulin, gamma-globulin; AST, aspartate aminotransferase; ALT, alanine aminotransferase; AST, ALT ratio, aspartate aminotransferase:alanine aminotransferase ratio; AlkP, alkaline phosphatase*.

Dietary VD_3_ did not influence the albumin/globulin ratio, α-1- and α-2-globulin, β-globulin, γ-globulin, ALT levels, the AST:ALT ratio, and alkaline phosphatase level. Besides, the serum protein fractions and liver index leakage enzymes are not affected by the interaction between the two variables.

It was found that increasing VD_3_ to a 1,000 and 1,200-IU/kg diet substantially increased total protein and globulin, compared to an 800 and 1,000 IU/kg diet, increased serum albumin compared to an 800 IU/kg diet. The immunomodulatory, anti-inflammatory, and anti-coccidia activity of VD_3_ or its metabolites have been reported in chickens (20, 23–26, 51, 52) and chicken cells (25, 27, 28). The antibody is proteomic in nature, and the increase in total protein and globulin levels supported the hypothesis that increasing Ca, and VD_3_ improves the immunity of laying hens, perhaps due to the protection from bone disease [rickets, osteoporosis, and cage layer fatigue (20, 41, 53)]. VD has an essential task in maintaining immunity and communication between the adaptive and innate immunity systems by affecting vitamin receptors of VD and activating enzymes (20, 54, 55).

The interaction effect indicates that the highest β-globulin was seen in groups fed 3.5 and 4% Ca, supplemented with 1,000 and 1,200 IU VD_3_, respectively, while the lowest values were from hens fed 4 and 4.5% Ca, supplemented with 1,000 and 800 IU VD_3_, respectively.

The γ-globulin, the material antibody, was significantly higher in hens fed 4.5% Ca than in hens fed 4% Ca when the unsupplemented groups were compared. Besides, supplementation of 4% Ca with 1,200 IU VD_3_ significantly decreased γ-globulin, compared to 1,000 IU VD_3_ supplementing the same Ca concentration. Also, supplementation of 4.5% Ca with 1,000 and 1,200 IU VD_3_ significantly decreased serum γ-globulin compared to 800 IU supplementation of the same Ca level.

### Immunoglobulin and Antibody Titer

Table 7 shows the effect of different dietary Ca and/or VD_3_ on blood plasma antioxidant enzymes and antibody titer. There was no effect of Ca level on most of the evaluated immune responses and antioxidant indices, except for serum IgG and IgA, plasma estrogen, and catalase (CAT). The results indicate that increasing Ca levels to 4 and 4.5% similarly increased IgG compared to 3.5% and raising the Ca level caused a gradual increase in serum IgA. Dietary Ca concentration affects plasma estrogen and Ca and VD_3_ influence CAT, but the effect was confounded by the significant interaction between dietary Ca and VD_3_ levels.

**Table 7:** Blood serum immunity parameters and some hormones of brown egg layers fed three levels of Ca supplemented with three levels of VD_3_ from 60 to 72 wk of age.

**Treatments**	**IgG, mg/dl**	**IgM, mg/dl**	**IgA, mg/dl**	**HIAI, Log2**	**HIND, Log2**	**Estrogen, Pg/ml**	**PTH, ng/ml**	**MDA, nmol/ml**	**TAC, nmol/ml**	**MDA/TAC Ratio**	**SOD, U/ml/h**	**CAT, U/ml/h**
**Calcium level**												
3.5% Ca	464^b^	134	154^c^	2.33	3.33	162^b^	2.17	21.9	1.75	12.8	76.1	47.2^c^
4.0% Ca	533^a^	131	165^b^	2.13	3.80	190^a^	2.12	19.1	1.76	11.0	74.5	54.0^b^
4.5% Ca	548^a^	137	175^a^	2.40	3.53	186^a^	2.17	19.5	1.63	12.2	80.8	57.6^a^
**VD**_**3**_ **level**												
800 IU	512	135	165	2.40	3.60	177	2.21	21.1	1.68	12.9	74.4	52.8^b^
1,000 IU	511	135	166	2.20	3.53	173	2.09	19.9	1.66	12.1	78.6	52.5^b^
1,200 IU	522	131	163	2.27	3.53	189	2.16	19.6	1.79	11.0	78.4	53.6^a^
**Interaction Ca × VD**_**3**_ **effect**												
3.5 800	407	135	151	2.20	3.00	134^b^	2.40	23.8	1.59	15.3	75.6	41.2^b^
3.5 1,000	485	136	155	2.20	3.60	175^a^^b^	2.09	21.5	1.79	12.1	75.4	51.2^a^^b^
3.5 1,200	499	132	156	2.60	3.40	177^a^^b^	1.99	20.4	1.85	11.1	77.2	49.3^a^^b^
4.0 800	542	130	164	2.40	4.20	199^a^	1.89	19.1	1.80	10.9	70.5	57.2^a^
4.0 1,000	500	126	165	2.20	3.60	175^a^^b^	2.17	19.6	1.71	11.6	77.9	48.4^a^^b^
4.0 1,200	558	137	167	1.80	3.60	196^a^	2.29	18.6	1.76	10.6	75.1	56.5^a^
4.5 800	586	140	179	2.60	3.60	196^a^	2.32	20.3	1.65	12.8	77.1	59.8^a^
4.5 1,000	548	142	179	2.20	3.40	169^a^^b^	2.02	18.5	1.49	12.6	82.4	57.9^a^
4.5 1,200	510	128	165	2.40	3.60	194^a^	2.18	19.6	1.76	11.2	83.0	55.8^a^
**Statistical variance**												
SEM	33.1	5.1	5.2	0.245	0.389	10.4	0.221	1.46	0.092	1.15	3.18	2.57
***P*****-values**												
Ca	0.008	0.391	0.002	0.391	0.348	0.005	0.943	0.055	0.201	0.166	0.054	0.001
VD_3_	0.901	0.817	0.658	0.599	0.971	0.166	0.832	0.404	0.197	0.123	0.201	0.001
Interaction	0.097	0.178	0.318	0.289	0.574	0.016	0.428	0.743	0.255	0.389	0.786	0.001

a,b,c *Means in the same column with different superscripts are significantly different (P < 0.05). SEM, standard error of the means; IgG, immunoglobulin G; IgM, immunoglobulin M; IgA, immunoglobulin A; HIAI, hemagglutination-inhibition test for avian influenza; HIND, hemagglutination-inhibition test for Newcastle disease virus; PTH, parathyroid hormone; MDA, malondialdehyde; TAC, total antioxidant capacity; SOD, superoxide dismutase; CAT, catalase*.

The lack of significant effects of VD_3_ on most immune responses (the type of globulin, immunoglobulins, and antibody titer), rather than total serum protein, albumin, and globulin, may indicate that the control diet supplemented with 800 IU, or a total VD_3_ of 3,800 IU, contains adequate VD_3_ to maintain the basic immune function. This may offset the effect of supplemented cholecalciferol on cellular and humoral immunity. This was further established by the lack of bone disease such as osteoporosis and cage layer fatigue in laying hens kept in cages under hot weather conditions. Such disorders indicate inadequate dietary VD_3_ (1, 20, 26).

In the literature, VD_3_ is reported to have many properties, such as antioxidant, immunomodulatory, anti-inflammatory, antiviral, antibacterial, anti-allergy, and cancer prevention activities (10–12). In poultry, the role of VD_3_ in Ca and Pi metabolism is fundamental for the development of bone and eggshell formation. In this regard, Rodriguez-Lecompte et al. (26) demonstrated that both VD_3_ and 25-(OH) D have strong immunomodulatory effects, such as increased positive helper T cell (Th2) response and autophagy. Laying hens kept under high environmental temperatures, such as those observed herein, are highly susceptible to infectious diseases, due to low immunity (49). Aslam et al. (56) found that VD deficiency decreases the cellular immune response in broilers. The literature reports that VD_3_ or 25-hydroxycholecalciferol has an anti-inflammatory activity in bird immune cells following the administration of lipo-oligosaccharides [LPSs (20, 57–60)]. Despite this, little attention has been paid to the influence of dietary VD_3_ supplementation or its deficiency on immune response and blood biochemistry of laying hens challenged with LPSs, or the mechanisms of action, although the anti-cancer and anti-inflammatory effects of VD_3_ are well-documented (17, 61–64).

### Plasma Hormones

The effect of Ca level and interaction between Ca level and VD_3_ was seen on levels of plasma estrogen (Table 7). It is clear that increasing Ca level from 3.5 to 4 or 4.5% increased plasma estrogen. Plasma PTH was not influenced by dietary Ca and/or VD_3_ concentrations. This indicates that the control diet (3.5% Ca and 3,800 IU VD_3_) contains adequate Ca and VD_3_ to maintain the normal level of PTH, which, together with 1,25-(OH)_2_D_3_, and controls calcium absorption in the digestive canal and calcium resorption from the bones and excretion to maintain Ca balance (1, 21, 26).

It was found that increasing Ca levels to 4 and 4.5% within the unsupplemented groups significantly, and similarly, increased plasma E_2_ compared to the 3.5% level. There were no significant changes in plasma estrogen within each Ca level or across different levels of Ca as a result of supplementation with VD_3_ when the corresponding levels were compared. The lowest E_2_ level was in hens fed 3.5% Ca supplemented with 800 IU of VD_3_, while the highest was from hens fed 4 and 4.5% Ca, supplemented with 800 and 1,200 IU of VD_3._ These findings demonstrate that a Ca level of 4% was adequate when supplemented with 800 IU of VD_3_ to enhance E_2_. The metabolism of Ca in laying hens is controlled by E_2_, PTH, and calcitonin (5, 21), and the increase in E_2_ was associated with increasing performance and eggshell quality (Table 7).

### Antioxidant Status

No effect was found on most of the antioxidant indices (TAC, MDA, and SOD) with Ca levels, except for CAT. Dietary Ca and VD_3_ concentration affected plasma CAT, but the effect was confounded by the significant interaction between dietary Ca and VD_3_ levels (Table 7). It was found that increasing the VD_3_ level to 1,000 and 1,200 IU in the 3.5% Ca group increased CAT to some extent. There no significant difference in CAT levels among different VD_3_ levels or within or between different Ca levels. These results show that the impact of VD_3_ on CAT depends on the dietary Ca concentration. Hence, antioxidant enzymes such as CAT need an adequate quantity of Ca and VD_3_ to be maintained for laying hens of 60–72 wk of age.

### Ovary and Reproductive Organs

The results of the relative weight of ovary and reproductive organs as affected by different Ca and VD_3_ concentrations are presented in Table 8.

**Table 8:** The length of parts of the female reproductive system of brown egg layers fed three levels of Ca supplemented with three levels of VD_3_ from 60 to 72 wk of age.

**Treatments**	**Ovary, %**	**Oviduct, cm**	**Infundibulum, %**	**Magnum, %**	**Isthmus, %**	**Uterus, %**
**Calcium level**						
3.5% Ca	59.2^b^	68.6^c^	8.42	51.6^a^	19.4^b^	12.0
4.0% Ca	62.8^a^	73.4^b^	7.95	50.6^b^	20.4^a^	12.5
4.5% Ca	62.4^a^	75.3^a^	8.09	50.4^b^	20.7^a^	12.2
**VD**_**3**_ **level**						
800 IU	68.6^c^	70.7^b^	8.30	51.2	19.9^b^	12.2
1,000 IU	73.5^b^	73.1^a^	8.26	51.2	19.6^b^	12.3
1,200 IU	75.3^a^	73.5^a^	7.90	50.3	21.0^a^	12.2
**Interaction Ca × VD**_**3**_ **effect**						
3.5 800	52.4^b^	64.3^d^	8.99	53.4^a^	17.7^b^	11.6
3.5 1,000	62.5^a^	69.6^c^	8.43	51.2^b^	19.2^a^	12.5
3.5 1,200	62.9^a^	71.9^b^^c^	7.83	50.3^b^	21.1^a^	11.9
4.0 800	62.2^a^	73.9^a^^b^	7.80	49.8^b^	21.1^a^	12.7
4.0 1,000	64.2^a^	73.4^a^^b^	8.27	50.9^b^	19.1^a^^b^	12.6
4.0 1,200	61.7^a^	73.0^a^^b^	7.76	51.0^b^	21.1^a^	12.1
4.5 800	60.7^a^	74.1^a^^b^	8.08	50.2^b^	20.8^a^	12.2
4.5 1,000	63.5^a^	76.3^a^	8.07	51.4^b^	20.4^a^	11.9
4.5 1,200	62.8^a^	75.6^a^	8.12	49.6^b^	20.9^a^	12.6
**Statistical variance**						
SEM	1.16	0.813	0.293	0.575	0.487	0.364
***P*****-values**						
Ca	0.001	0.001	0.146	0.025	0.006	0.327
VD_3_	0.001	0.034	0.218	0.105	0.003	0.848
Interaction	0.001	0.001	0.190	0.004	0.002	0.139

a,b,c,d *Means in the same column with different superscripts are significantly different (P < 0.05). SEM, standard error of the means*.

The Ca level in diet had an impact (*P* < 0.05) on the percentage of the ovary, magnum, the isthmus, and the absolute oviduct length (cm), and VD_3_ concentrations showed a marked influence on the relative weight of the ovary and isthmus and the oviduct length. These impacts were confounded by the interaction between Ca and VD_3_.

The results of the interaction indicate that increasing Ca levels with the groups supplemented with 800 IU of VD_3_ significantly increased the relative weight of ovary and isthmus and absolute length of the oviduct, compared to the control diet containing 3.5% Ca and 3,800 IU of total VD_3_, but decreased the magnum percentage. It was found that elevating the VD_3_ concentrations to 1,000 (4,000 IU as total VD_3_) and 1200 IU (4,200 IU as total VD_3_) within the 3.5% Ca level similarly elevated the relative weights of ovary and isthmus and the absolute oviduct length, but decreased the relative weight of the magnum. These changes in ovary and oviduct, except for the magnum, reflected the positive changes in E_2_ and enhanced the laying performance of hens fed 3.5% Ca when supplemented with 1,000 or 1,200 IU of VD_3_.

In conclusion, increasing dietary Ca levels in laying hens up to 4% during the late production phase could be a useful tool to improve laying performance, eggshell quality, Haugh unit, and physiological and immunological status. Besides, supplementing 3.5% Ca diets with 1,000 IU of VD_3_ or a total 4,000 IU/kg diet VD_3_ improved performance of hens fed 3.5% Ca level during late stage of production (60–72 wk of age), showing that the impact of VD_3_ depends on dietary Ca concentrations.

## Data Availability Statement

All datasets generated for this study are included in the article/supplementary material.

## Ethics Statement

The experimental procedures were approved by King Abdulaziz University, Jeddah, Saudi Arabia under protocol number (DF-715-155-1441H) that recommends animal rights, welfare, and minimal stress and did not cause any harm or suffering to animals according to the Royal Decree number M59 in 14/9/1431H.

## Author Contributions

All authors listed have made a substantial, direct and intellectual contribution to the work, and approved it for publication.

## Conflict of Interest

The authors declare that the research was conducted in the absence of any commercial or financial relationships that could be construed as a potential conflict of interest.

## References

[1] Oluyemi JA Roberts FA Poultry Production in Warm Wet Climates. 2nd ed Ibadan: Spectrum Books Limited (2000).

[2] Pizzolante CC Saldanha ESPB Lagana C Kakimoto SK Toghashi CK Effect of calcium levels and limestone pareticle size on the egg quality of semi-heavy layers in their second produc-tion cycle. Braz J Poult Sci. (2009) 11:79–86. 10.1590/S1516-635X2009000200002

[3] Saunders-Blades JL Korver DR. Effect of hen age and maternal vitamin D source on performance, hatchability, bone mineral density, and progeny *in vitro* early innate immune function. Poult Sci. (2015) 94:1233–46. 10.3382/ps/pev002 25743414

[4] Keshavarz KA Comparison between cholecalciferol and 25-OH-cholecalciferol on performance and eggshell quality of hens fed different levels of calcium and phosphorus. Poult Sci. (2003) 82:1415–22. 10.1093/ps/82.9.1415 12967255

[5] Roland DA Egg shell quality III: calcium and phosphorus requirements of commercial Leghorns. Worlds Poult Sci J. (1986) 42:154–65. 10.1079/WPS19860012

[6] Al-Batshan HA Scheideler SE Black BL Garlich JD Anderson KE. Duodenal calcium-uptake, femur ash, and eggshell quality decline with age and increase following molt. Poult Sci. (1994) 73:1590–6. 10.3382/ps.0731590 7816734

[7] Saunders-Blades JL MacIsaac JL Korver DR Anderson DM. The effect of calcium source and particle size on the production performance and bone quality of laying hens. Poult Sci. (2009) 88:338–53. 10.3382/ps.2008-00278 19151349

[8] Gregory NG Wilkins LJ. Broken bones in domestic fowl: handling and processing damage in end-of-lay battery hens. Br Poult Sci. (1989) 30:555–62. 10.1080/00071668908417179 2819499

[9] Marie P Labas V Brionne A Harichaux G Hennequet-Antier C Nys Y. Quantitative proteomics and bioinformatic analysis provide new insight into protein function during avian eggshell biomineralization. J Proteomics. (2015) 113:178–93. 10.1016/j.jprot.2014.09.024 25284052

[10] Marie P Labas V Brionne A Harichaux G Hennequet-Antier C Rodriguez-Navarro AB. Quantitative proteomics provides new insights into chicken eggshell matrix protein functions during the primary events of mineralisation and the active calcification phase. J Proteomics. (2015) 126:140–54. 10.1016/j.jprot.2015.05.034 26049031

[11] Attia YA Al-Harthi MA Shiboob MM Evaluation of quality and nutrient contents of table eggs from different sources in the retail market. Ital J Anim Sci. (2014) 13:3269 10.4081/ijas.2014.3294

[12] Keshavarz K Scott ML Blanchard J The effect of solubility and particle size of calcium sources on shell quality and bone mineralization. J Appl Poult Res. (1993) 2:259–67. 10.1093/japr/2.3.259

[13] Clunies M Park D Leeson S. Calcium and phosphorus metabolism and eggshell formation of hens fed different amounts of calcium. Poult Sci. (1992) 71:482–9. 10.3382/ps.0710482 1561214

[14] Wallner-Pendleton E Scheideler SE The Influence of Varying Levels of Calcium and Vitamin D_3_ in the Mature Laying Hen's Diet: Effect on Egg Production, Shell Quality, Bone Ash and Urolithiasis. The Nebraska Poultry Report. University of Nebr Coop Ext EC, Lincoln (1996).

[15] Roush WB Mylet M Rosenberger JL Derr J Investigation of calcium and available phosphorus requirement of laying hens by response surface methodology. Poult Sci. (1986) 65:964–70. 10.3382/ps.0650964

[16] Tierzucht L Layers LBC Lohaman-Brown-Classic Layers Management–Guide Cage Housing. (2016). Available online at: https://www.ltz.de/ (accessed March 20, 2018).

[17] Dale N National Research Council Nutrient Requirements of Poultry 9th Revised Edition. Washington, DC: National Academy Press (1994).

[18] Guidelines 2016 for Domestic Animals Health Nutrition Materials DSM Vitamin Supplementation Guidelines 2016 for Animal Nutrition. (2016). p. 1–26. Available online at: https://www.dsm.com/content/dam/dsm/anh/en_US/documents/Vitamin_Supp_Guidelinespdf.?download=a1debc5c-f2e3-43c5-b782-64b7257f55b21584479483674 (accessed March 25, 2019).

[19] Stevens VI Blair R Salmon RE. Effects of VD_3_, calcium, and phosphorus on growth and bone development of market turkeys. Poult Sci. (1984) 63:1571–85. 10.3382/ps.0631571 6091081

[20] Geng Y Ma Q Wang ZD Guo Y. Dietary vitamin D3 supplementation protects laying hens against lipopolysaccharide-induced immunological stress. Nutr Metab. (2018) 15:58. 10.1186/s12986-018-0293-8 30116287 PMC6086064

[21] Scott ML Nesheim MC Young RJ Nutrition of the Chicken. Ithaca, NY: Scott ML and Associates (1982).

[22] Soares JH Jr Kerr JM Gray RW. 25-Hydroxycholicalciferol in poultry nutrition. Poult Sci. (1995) 74:1919–34. 10.3382/ps.0741919 8825582

[23] Overbergh L Decallonne B Valckx D Verstuyf A Depovere J Laureys J. Identification and immune regulation of 25-hydroxyvitamin D-1-alpha-hydroxylase in murine macrophages. Clin Exp Immunol. (2000) 120:139–46. 10.1046/j.1365-2249.2000.01204.x 10759775 PMC1905630

[24] Gorman S Buckley AG Ling KM Berry LJ Fear VS Stick SM. Vitamin D supplementation of initially vitamin D-deficient mice diminishes lung inflammation with limited effects on pulmonary epithelial integrity. Physiol Rep. (2017) 15:e13371. 10.14814/phy2.13371 28774952 PMC5555896

[25] Daghir NJ Poultry Production in Hot Climates. 2nd ed Cambridge, MA: CABI (2008).

[26] Rodriguez-Lecompte JC Yitbarek A Cuperus T Echeverry H van Dijk A. The immunomodulatory effect of vitamin D in chickens is dose-dependent and influenced by calcium and phosphorus levels. Poult Sci. (2016) 95:2547–56. 10.3382/ps/pew186 27252374

[27] Manolagas SC Provvedini DM Tsoukas CD Interactions of 1, 25-dihydroxy VD3 and the immune system. Mol Cell Endocrinol. (1985) 43:113–22. 10.1016/0303-7207(85)90074-7 3000847

[28] H & N International Brown Nick, Brown Egg Layers. New Management Guide – H&N International GmbH. (2016). p. 76 Available online at: https://www.hn-int.com/eng-wAssets/docs/managementguides/Layers-englisch/HN_MG_Brown-Nick_EN.pdf (accessed May 25, 2019).

[29] AOAC Official Methods of Analysis. Washington, DC: Association of Official Analytical Chemists (2004).

[30] Burke WH Attia YA. Molting single comb white leghorns with the use of the lupron depot formulation of leuprolide acetate. Poult Sci. (1995) 73:1226–32. 10.3382/ps.0731226 7971664

[31] Attia YA Burke WH Yamani KA. Response of broiler breeder hens to forced molting by hormonal and dietary manipulations. Poult Sci. (1994) 73:245–58. 10.3382/ps.0730245 8146072

[32] Attia YA Burke WH Yamani KA Jensen LS Energy allotments and performance of broiler breeders 2- females. Poult Sci. (1995) 74:261–70. 10.3382/ps.0740261 7724449

[33] Stefanello C Santos TC Murakami AE Martins EN Carneiro TC. Productive performance, eggshell quality, and eggshell ultrastructure of laying hens fed diets supplemented with organic trace minerals. Poult Sci. (2014) 93:104–13. 10.3382/ps.2013-03190 24570429

[34] Spectrum, Corp for Biotech SAE, Egypt Spectrum Diagnostic Kits. (2016) Available online at: http://www.spectrum-diagnostics.com/new/

[35] Attia YA Al-Khalaifah HS Abd El-Hamid HE Al-Harthi MA El-shafey AA Effect of different levels of multi-enzymes on immune response, blood hematology and biochemistry, antioxidants status and organs histology of broiler chicks fed standard and low-density diets. Front Vet Sci. (2020) 6:510 10.3389/fvets.2019.00510 32195272 PMC7015166

[36] Attia YA Al-Khalifa H Ibrahim MS Abd Al-Hamid AE Al-Harthi MA El-Naggar Asmaa Blood hematological and biochemical constituents, antioxidant enzymes, immunity and lymphoid organs of broiler chicks supplemented with propolis, bee pollen and mannan oligosaccharides continuously or intermittently. Poult Sci. (2017) 96:4182–92. 10.3382/ps/pex173 29053876

[37] SAS. SAS® User's Guide Statistics Version. 10th ed. Cary, NC: SAS Institute Inc (2004).

[38] Rao KS Roland DA. Influence of dietary calcium level and particle size of calcium source on *in vivo* calcium solubilization by commercial leghorns. Poult Sci. (1989) 68:1499–505. 10.3382/ps.0681499 2608615

[39] Zhang B Coon CN. The relationship of calcium intake, source, size, solubility *in vitro* and *in vivo*, and gizzard limestone retention in laying hens. Poult Sci. (1997) 76:1702–6. 10.1093/ps/76.12.1702 9438285

[40] Nascimento GR Murakami AE Guerra AFQM Ospinas-Rojas IC Ferreira MFZ Fanhani JC Effect of different vitamin D sources and calcium levels in the diet of layers in the second laying cycle. Braz J Poult Sci. (2014) 16:37–42. 10.1590/1516-635x160237-42

[41] Elaroussi MA Forte LR Eber SL Biellier HV. Calcium homeostasis in the laying hen 1 age and dietary calcium effects. Poult Sci. (1994) 73:1581–9. 10.3382/ps.0731581 7816733

[42] Duran MR Chen C Kim WK Effects of vitamin d and calcium for the prevention of osteoporosis at various stages of life of laying hens-review. Inter J Poult Sci. (2018) 17:405–9. 10.3923/ijps.2018.405.409

[43] Sauveur B Lésions osseuses et articulaires des pattes des volailles: rôles de l'alimentation. Prod Anim. (1988) 11:35–45.

[44] Nys Y Gautron J Structure and formation of the eggshell. In: Huopalahti R López-Fandiño R Anton M Schade R, editors. Bioactive Egg Compounds. Berlin: Springer (2007). p. 99–102.

[45] El-Safty SA Stepwise regression analysis for ultrastructural measurements of eggshell quality in two local breeds of chicken. Egypt Poult Sci. (2004) 24:189–203.

[46] Fathi MM Zein El-Dein A El-Safty SA Radwan LM Using scanning electron microscopy to detect the ultrastructural variations in eggshell quality of Fayoumi and Dandarawi chicken breeds. Int J Poult Sci. (2007) 6:236–41. 10.3923/ijps.2007.236.241

[47] Susanna K Fröhlich E Gebhardt-Henrich GS Schäublin H Pfulg A Zweifel R Effects of dietary supplementation with synthetic vitamin D3 and 25-hydroxycholecalciferol on blood calcium and phosphate levels and performance in laying hens. ArchGeflügelk. (2011) 75:179–84.

[48] Baimbridg KG Taylor TG The role of calcitonin in controlling hypercalcaemia in the domestic fowl *(Gallus domesticus)*. Comp. Bioch. Phys. Part A Phys. (1981) 68:647–51. 10.1016/0300-9629(81)90372-8

[49] Rosemary L Walzem RJ Hansen DL Williams RL. Hamilton estrogen induction of VLDLy assembly in egg-laying hens. J Nut. (1999) 129:467S−72S. 10.1093/jn/129.2.467S 10064311

[50] Garlich J Brake J Parkhurst CR Thaxton JP Morgan GW. Physiological profile of caged layers during one production year, molt, and postmolt: egg production, egg shell quality, liver, femur, and blood parameters. Poult Sci. (1984) 63:339–33. 10.3382/ps.0630339 6709570

[51] Barreda DR Konowalchuk JD Rieger AM Wong ME Havixbeck JJ. Triennial growth symposium–novel roles for vitamin D in animal immunity and health. J Anim Sci. (2014) 92:930–8. 10.2527/jas.2013-7341 24665105

[52] Attia YA Abd El-Hamid AE Abedalla AA Berika MA El-Gandy MF Sahin K Effect of betaine, vitamin C, and vitamin E on egg quality, hatchability, and markers of liver and renal functions in dual-purpose breeding hens exposed to chronic heat stress. Europ Poult Sci. (2018) 82 10.1399/eps2017171

[53] Suaini NH Zhang Y Vuillermin PJ Allen KJ Harrison LC. Immune modulation by vitamin D and its relevance to food allergy. Nutrition. (2015) 7:6088–108 10.3390/nu7085271 26225992 PMC4555110

[54] Adams JS Hewison M. Unexpected actions of vitamin D: new perspectives on the regulation of innate and adaptive immunity. Nat Clin Pract Endocrinol Metab. (2008) 4:80–90. 10.1038/ncpendmet0716 18212810 PMC2678245

[55] Li YC Chen YZ Liu WC Thadhani R. MicroRNA-mediated mechanism of vitamin D regulation of innate immune response. J Steroid Biochem Mol Biol. (2014) 144PA:81–6. 10.1016/j.jsbmb.2013.09.014 24103701 PMC3976893

[56] Aslam SM Garlich JD Qureshi MA. Vitamin D deficiency alters the immune responses of broiler chicks. Poult Sci. (1998) 77:842–9. 10.1093/ps/77.6.842 9628532

[57] Lu L Li SM Zhang L Liu XQ Li DY Zhao XL. Expression of beta defensins in intestines of chickens injected with vitamin D3 and lipopolysaccharide. Genet Mol Res. (2015) 14:3330–7. 10.4238/2015.April.13.12 25966099

[58] Morris A Shanmugasundaram R Lilburn MS Selvaraj RK. 25- hydroxycholecalciferol supplementation improves growth performance and decreases inflammation during an experimental lipopolysaccharide injection. Poult Sci. (2014) 93:1951–6. 10.3382/ps.2014-03939 24931970

[59] Morris A Shanmugasundaram R McDonald J Selvaraj RK. Effect of *in vitro* and *in vivo* 25-hydroxyvitamin D treatment on macrophages, T cells, and layer chickens during a coccidia challenge. J Anim Sci. (2015) 93:2894–903. 10.2527/jas.2014-8866 26115276

[60] Shojadoost B Behboudi S Villanueva AI Brisbin JT Ashkar AA Sharif S. Vitamin D3 modulates the function of chicken macrophages. Res Vet Sci. (2015) 100:45–51. 10.1016/j.rvsc.2015.03.009 25814176

[61] Krishnan AV Feldman D. Mechanisms of the anti-cancer and anti-inflammatory actions of vitamin D. Annu Rev Pharmacol Toxicol. (2011) 51:311–36. 10.1146/annurev-pharmtox-010510-100611 20936945

[62] Xu S Chen YH Tan ZX Xie DD Zhang C Xia MZ. Vitamin D_3_ pretreatment alleviates renal oxidative stress in lipopolysaccharide-induced acute kidney injury. J Steroid Biochem Mol Biol. (2015) 152:133–41. 10.1016/j.jsbmb.2015.05.009 26013770

[63] Di Rosa M Malaguarnera M Nicoletti F Malaguarnera L. Vitamin D_3_: a helpful immuno-modulator. Immunology. (2011) 134:123–39. 10.1111/j.1365-2567.2011.03482.x 21896008 PMC3194221

[64] Zhang L Lu L Li S Zhang G Ouyang L Robinson K. 1,25-Dihydroxyvitamin-D_3_ induces avian beta-Defensin gene expression in chickens. PLoS ONE. (2016) 11:e0154546. 10.1371/journal.pone.0154546 27135828 PMC4852925

